# Genetic analysis of varicella-zoster virus in the aqueous humor in uveitis with severe hyphema

**DOI:** 10.1186/s12879-017-2518-2

**Published:** 2017-06-15

**Authors:** Mayumi Hosogai, Yoko Nakatani, Kensuke Mimura, Shoji Kishi, Hideo Akiyama

**Affiliations:** 1 Department of Ophthalmology, Gunma University Graduate School of Medicine, 3-39-22 Showa-machi, Maebashi, Gunma 371-8511 Japan; 2 Department of Virology and Preventive Medicine, Gunma University Graduate School of Medicine, 3-39-22 Showa-machi, Maebashi, Gunma 371-8511 Japan

**Keywords:** Varicella-zoster virus, Herpes zoster ophthalmicus, Zoster sine herpete, Hyphema, Uveitis, Polymerase chain reaction, Genotype, Single nucleotide polymorphism

## Abstract

**Background:**

Genetic variations have been identified in the genome of varicella-zoster virus (VZV) strains using vesicle fluid, varicella scabs and throat swab samples. We report a rare case of VZV-associated uveitis with severe hyphema, which was immediately diagnosed by polymerase chain reaction (PCR) using the aqueous humor, in which we were able to analyze the VZV genotype for the first time.

**Case presentation:**

A 16-year-old Japanese boy was referred to our hospital with a 20-day history of unilateral anterior uveitis and 11-day history of hyphema. At presentation, details of the iris, the iridocorneal angle, and the fundus were not visible due to the severe hyphema. Serum anti-VZV IgG and anti-VZV IgM were elevated, and 1.61 × 10^9^ copies/mL of VZV-DNA were detected by real-time PCR using the aqueous humor. As there were no eruptions on his face or body, we diagnosed zoster sine herpete and started intravenous administration of prednisolone and acyclovir. The hyphema completely disappeared 2 weeks after presentation, while sectorial iris atrophy and mild periphlebitis of the fundus became gradually apparent. Anterior inflammation and periphlebitis gradually improved and VZV-DNA in the aqueous humor was reduced to 1.02 × 10^6^ copies/mL at 4 weeks after presentation. Examination by slit lamp microscope revealed no inflammation after 5 months, and VZV-DNA could no longer be detected in the aqueous humor. Serum anti-VZV IgG and anti-VZV IgM also showed a gradual decrease along with improvement in ocular inflammation. The genetic analysis of multiple open reading frames and the R5 variable repeat region in the VZV genes, using DNA extracted from the aqueous humor at presentation, showed that the isolate was a wild-type clade 2 VZV strain (prevalent in Japan and surrounding countries) with R5A allele and one SNP unique to clade 1 (both are major types in Europe and North America).

**Conclusions:**

VZV-associated uveitis may develop hyphema that obscures ocular inflammation, thus PCR analysis using the aqueous humor is the key investigation necessary for the diagnosis. The measurement of VZV-DNA copies by real-time PCR would be useful for evaluation of therapeutic effects. We could amplify and analyze VZV genotype using the aqueous humor including a very large number of VZV-DNA copies (1.61 × 10^9^ copies/mL).

## Background

Varicella-zoster virus (VZV) is highly contagious and globally ubiquitous. The primary infection results in varicella (chickenpox), which usually occurs early in life. Subsequently, the virus establishes a lifelong latent infection in the sensory nerve ganglia, which reactivates to cause herpes zoster [1].

The VZV genome consists of about 125,000 bases of linear, double-stranded DNA with ≧ 70 open reading frames (ORFs). Since the 1990s, a number of genetic variations have been identified in the genome of VZV strains by the use of molecular techniques such as sequencing, restriction fragment length polymorphism (RFLP) and single nucleotide polymorphism (SNP) [2–9]. In 2010, Breuer et al. summarized the previous nomenclature systems and proposed a novel nomenclature for VZV: clade 1–5 and two putative clades (VI and VII) [10]. In these previous reports, VZV-DNA was extracted from vesicle fluid, varicella scabs or throat swab samples.

Herpes zoster involvement in the ophthalmic division of the first branch of the trigeminal nerve is called herpes zoster ophthalmicus (HZO). HZO without skin lesions is known as zoster sine herpete (ZSH). VZV-associated uveitis may develop in both HZO and ZSH [11]. VZV anterior uveitis is characterised by mutton-fat keratic precipitates, trabecular meshwork pigmentation, ocular hypertension, iris atrophy and distorted pupil. Uveitis sometimes causes hyphema which hides these typical findings [12, 13]. Few cases of severe hyphema as a complication following VZV-associated uveitis have been reported [14–16]. These cases were diagnosed by clinical findings (e.g., facial skin eruptions) or serum and aqueous humor levels of anti-VZV IgG; however, a confirmed diagnosis is difficult and time-consuming.

Here, we report a rare case of VZV-associated uveitis with severe hyphema, early diagnosed by multiplex polymerase chain reaction (PCR) using the aqueous humor, in which we were able to amplify and analyze the VZV genotype for the first time.

## Case presentation

**Fig. 1:**
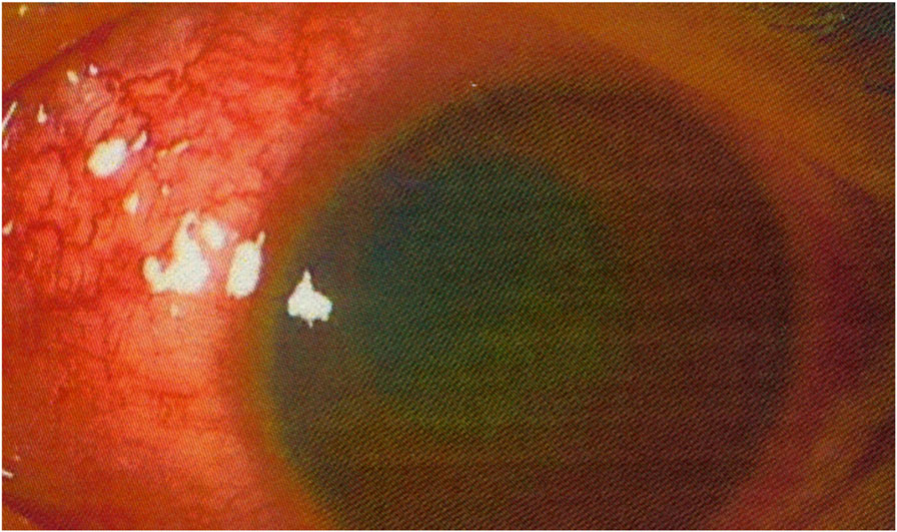
An anterior photograph of the left eye taken at presentation. Ciliary injection, corneal edema and severe hyphema filling the anterior chamber can be observed. Due to the severe hyphema, details of the iris, the iridocorneal angle and the fundus were not visible

A 16-year-old Japanese boy was referred to our hospital from an ophthalmology clinic because of severe hyphema in the left eye and anterior uveitis that had persisted for 11 and 20 days, respectively. He was receiving topical treatment with 0.1% betamethasone, 1% atropine, 1.5% levofloxacin, and 0.5% timolol maleate in the left eye. There was no ocular history of trauma, inflammation, or medication. His family history and medical history for systemic diseases were unremarkable. At presentation, the best corrected visual acuity was 20/15 in the right eye and 20/2000 in the left eye. Intraocular pressure (IOP) was 12 mmHg in the right eye and 29 mmHg in the left eye. Slit lamp biomicroscopy of the left eye revealed ciliary injection, corneal edema, and severe hyphema filling the anterior chamber (Fig. 1). Due to the severe hyphema, details of the iris, the iridocorneal angle, and the fundus were not visible. No abnormalities were found in the right eye. B-mode echo examination of the left eye revealed no obvious abnormality. In order to exclude the possibility that the hyphema was caused by ocular ischemia, carotid ultrasound was performed, but no obstruction was observed. There was no difference in blood pressure between the two arms suggestive of ocular ischemia caused by Takayasu disease.

Routine blood tests revealed no abnormalities in blood cell counts, blood glucose level, C-reactive protein, immunoglobulins (IgG, IgA, and IgM), anti-nuclear antibody, or rheumatoid factor. However, anti-VZV IgG and anti-VZV IgM measured by enzyme-linked immunosorbent assays were elevated to >2600 mIU/mL (negative: < 50 mIU/mL) and 1: 672 (negative: < 1: 42), respectively. VZV-DNA was not detected in the plasma.

In addition to the laboratory blood tests, we performed an anterior chamber paracentesis of the left eye and extracted DNA from the aqueous humor sample using QIAamp DNA Mini Kit (Qiagen, Valencia, CA). To detect human herpes viruses 1 through 6, multiplex PCR was also performed. VZV-DNA was then detected by multiplex PCR and quantitative real-time PCR revealed 1.61 × 10^9^ copies/mL of VZV-DNA. He had not been vaccinated for VZV and there was history of chickenpox in early childhood. As there were no skin eruptions on his face or body, we diagnosed ZSH and excluded HZO. A detailed examination of the fundus was impossible because of hyphema, so we initiated intravenous administration of acyclovir (500 mg/day) and dexamethasone (6.6 mg/day) based on the possibility of acute retinal necrosis.

**Fig. 2:**
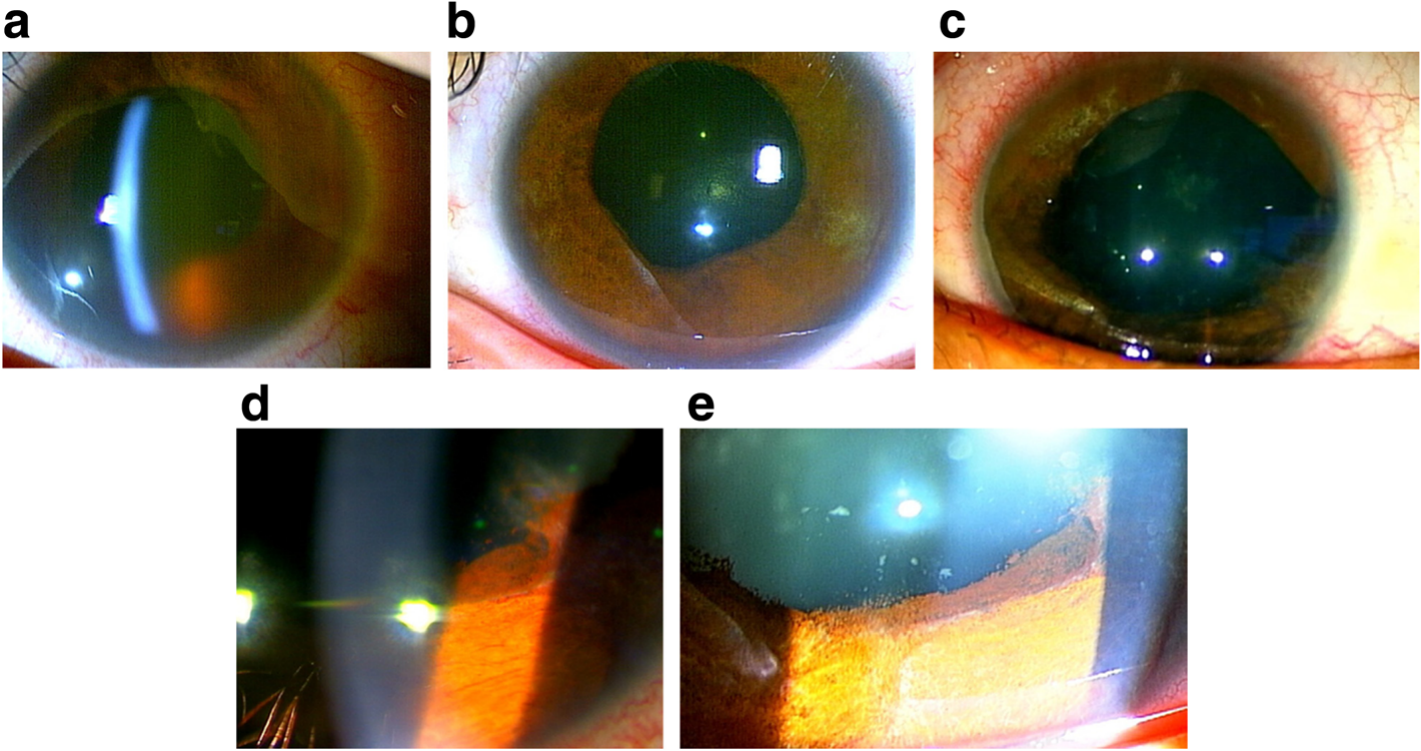
Anterior photographs of the left eye. **a** At 2 days after presentation, the hyphema had been partially absorbed and visibility of anterior chamber had improved. **b** At 2 weeks after presentation, the hyphema had completely disappeared and segmental iris atrophy was revealed. **c** At 4 months after presentation, segmental iris atrophy, distorted pupil, and posterior synechiae were observed. **d** At 4 months after presentation, rubeosis iridis emerged. **e** At 5 months after presentation, the rubeosis iridis had disappeared and iris atrophy was clearly observable

The hyphema was gradually absorbed and visibility of the anterior chamber improved, revealing inflammation of the anterior chamber and distortion of the pupil (Fig. 2a). A subconjunctival injection of dexamethasone (1 mg) was given in the left eye at 4 days after the initial visit in response to persisting ciliary injection and anterior inflammation. At six days after the initial visit, as the hyphema had diminished, allowing the fundus to be observed, and mild peripheral periphlebitis in the fundus was confirmed.

At two weeks after the initial visit, the hyphema had completely resolved and segmental iris atrophy, which is one of the characteristic ocular manifestations of VZV-associated uveitis, was visible (Fig. 2b). Visual acuity improved to 20/25 and the IOP was 9 mmHg in the left eye. As the periphlebitis also improved, the patient was discharged and the drip was changed to oral famciclovir (1500 mg/day) and prednisolone (30 mg/day). As the anterior inflammation and periphlebitis continued to gradually improve thereafter, oral prednisolone was reduced by 5 mg every week.

At 4 weeks after the initial visit, anti-VZV IgG and anti-VZV IgM had decreased to 2500 mIU/mL and 1: 168, respectively. VZV-DNA in the aqueous humor was reduced to 1.02 × 10^6^ copies/mL. At 2 months after presentation, there was no deterioration in ocular inflammation, so oral famciclovir was reduced to 750 mg/day. At 3 months after presentation, the ciliary injection, anterior inflammation, and periphlebitis had become very mild, and the serum anti-VZV IgG and anti-VZV IgM were further decreased to 1300 mIU/mL and 1: 42, respectively. Treatment was continued with oral famciclovir (750 mg/day) and prednisolone (5 mg/day).

At 4 months after presentation, as the ciliary injection and inflammation of the anterior chamber had worsened and rubeosis iridis emerged (Fig. 2c, d), a subtenon injection of triamcinolone (30 mg) was given to the left eye in addition to the topical and oral medications. After discussing the pros and cons of the therapy and receiving written informed consent, the patient received an intravitreal injection of bevacizumab (1.25 mg/0.05 ml).

At 5 months after presentation, visual acuity was 20/25 and the IOP was 10 mmHg in the left eye. The anterior inflammation and periphlebitis had almost completely resolved and the rubeosis iridis had disappeared (Fig. 2e). Slit lamp ophthalmoscopic examination revealed segmental iris atrophy, a distorted pupil, pigmented keratic precipitates, and posterior synechiae. As the retinal periphlebitis had also improved and VZV-DNA in the aqueous humor was not detected by PCR, the oral famciclovir was stopped. However, 5 mg of prednisone every other day and topical medication with 0.1% betamethasone were continued until 12 months after presentation.

**Fig. 3:**
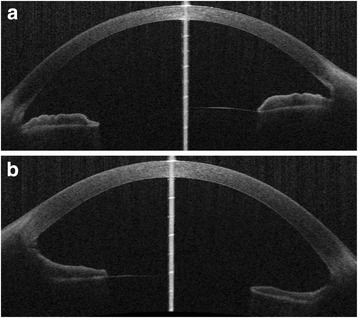
Anterior segment OCT images obtained from the CASIA OCT at the horizontal meridian. Thinning of the iris is observable in the left eye (**b**) compared with the right eye (**a**) at 14 months after presentation

14 months after the initial visit, visual acuity was 20/40 and the IOP was 12 mmHg in the left eye. There was no recurrence of severe inflammation, but a cataract had gradually developed. Anterior segment optical coherence tomography (OCT) images obtained from the CASIA (Tomey, Nagoya, Japan) revealed thinning of the iris in the left eye (Fig. 3). Serum anti-VZV IgG and anti-VZV IgM showed a gradual decrease to 670 mIU/mL and <1: 42, respectively.

### Genetic analysis of VZV

**Fig. 4:**
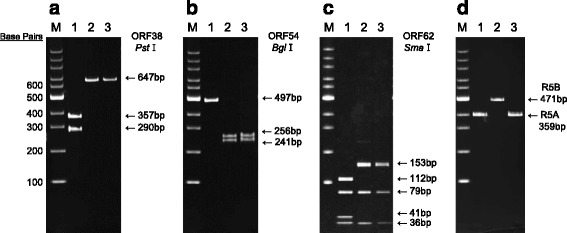
Genetic analysis of VZV-DNA detected in the aqueous humor. Polyacrylamide gel electrophoresis of the restriction digestion products showing: **a** *Pst*I digestion of ORF 38 amplified products, **b** *Bgl*I digestion of ORF 54 amplified products, and **c** *Sma*I digestion of ORF 62 amplified products. **d** DNA fragments after amplification of the R5 variable region by PCR. Lane M: 100-bp molecular weight ladder. The strain Ellen (Lane 1) and the strain Kawaguchi (Lane 2) were used to represent the American laboratory VZV and the Japanese wild-type VZV, respectively, as controls. Lane 3: DNA fragment from the aqueous humor of this patient at presentation

In order to compare the genotype of the VZV isolate from the aqueous humor of this patient to the previously reported genotypes of the isolates from vesicle fluid, varicella scabs or throat swab samples, we pursued additional genotypic characterization of the VZV strain using DNA extracted from the aqueous humor at the initial visit (1.61 × 10^9^ copies/mL of VZV-DNA). Attempts to sequence for VZV genotyping using DNA obtained at 4 weeks after presentation (1.02 × 10^6^ copies/mL) were unsuccessful due to the low viral load. First, we analyzed the RFLPs of ORF38 (*Pst*I), ORF54 (*Bgl*I), and ORF62 (*Sma*I) as previously described [6, 8, 9]. The strain Ellen [17, 18] and the strain Kawaguchi [19] were used to represent the American laboratory VZV and the Japanese wild-type VZV, respectively, as controls. Most VZV isolates in Europe and America, including strain Dumas [20], strain MLS [21], and strain Ellen, contain a *Pst*I restriction enzyme site in ORF38 (positive for the *Pst*I site: *Pst*I^+^). In this case, the PCR products of ORF38 (647 bp) lacked this restriction site (negative for the *Pst*I site: *Pst*I^−^) (Fig. 4a). The PCR products of ORF54 (497 bp) contained the *Bgl*I restriction enzyme site (*Bgl*I^+^) and yielded 2 fragments of 256 and 241 bp (Fig. 4b). The PCR products of the Oka vaccine (v-Oka) strain have three *Sma*I restriction enzyme sites in ORF62 and yield a set of 112, 79, 41, and 36 bp fragments (positive for the *Sma*I site: *Sma*I^+^) [8]. In this patient, the PCR products of ORF62 (268 bp) contained two restriction enzyme sites and were cleaved into a set of 153, 79, and 36 bp fragments (negative for the *Sma*I site: *Sma*I^−^) like most wild-type VZV strains (Fig. 4c). In summary, RFLPs analysis showed that the isolates from the aqueous humor of this patient were negative for the *Pst*I site (*Pst*I^−^), positive for the *Bgl*I site (*Bgl*I^+^), and negative for the *Sma*I site (*Sma*I^−^), which are typical of Japanese wild-type strains such as the parental Oka (p-Oka) strain and strain Kawaguchi (Table 1).

**Table 1:** Genotyping of VZV in the aqueous humor of this patient in reference to the published strain

VZV strain [Reference]	Origin country	Strain origin	ORF38 (*Pst*I)	ORF54 (*Bgl*I)	ORF62 (*Sma*I)	R5 type	clade [10]
Dumas [20]	Netherlands	Varicella	+	-	-	R5A	1
v-Oka [19]	Japan	Vaccine	-	+	+	R5B	2
p-Oka [19]	Japan	Varicella	-	+	-	R5B	2
MLS [21]	USA	Not available	+	+	-	R5A	5
Ellen [17, 18]	USA	Varicella	+	-	+	R5A	3
Kawaguchi [19]	Japan	Varicella	-	+	-	R5B	2
this case	Japan	Aqueous humor	-	+	-	R5A	2

The R5 variable region between ORF60 and ORF61 has also been shown to vary among different VZV strains. The R5 variable region was amplified by PCR and analyzed by electrophoresis as previously described [6]. This region contains a repeating unit composed of one 88-bp element and one 24-bp element, the copy number of which varied from one to three depending on the VZV strain [22]. The size of the PCR products can be calculated using the following formula, where n represents the number of repeating units:

$$ \mathrm{Size}\ \mathrm{of}\ \mathrm{the}\ \mathrm{PCR}\ \mathrm{products}\ \left(\mathrm{bp}\right)=24\mathrm{n}+88\ \left(\mathrm{n}+1\right)+159 $$

The PCR products of the R5A type are 359 bp (*n* = 1), those of the R5B type are 471 bp (*n* = 2), and those of the R5C type are 583 bp (*n* = 3). In this patient, R5A (mainly found in Europe and North America) was observed unlike Japanese strains (v-Oka, p-Oka, and Kawaguchi) (Fig. 4d and Table 1).

**Fig. 5:**
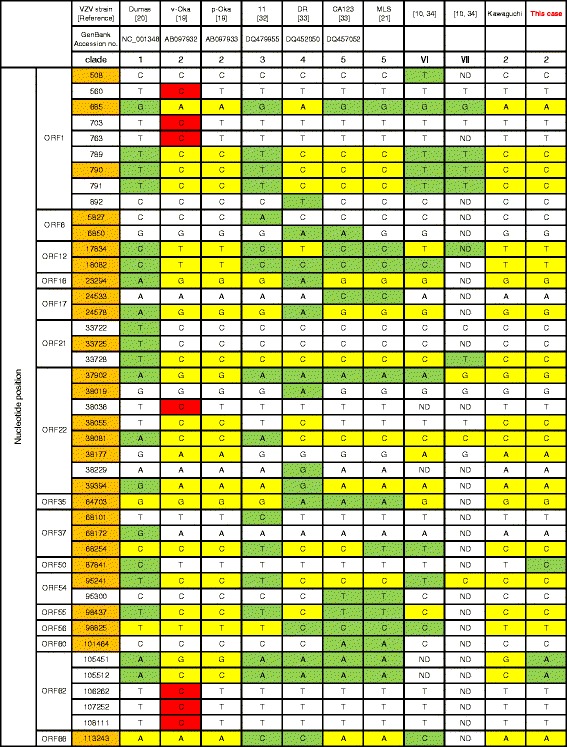
Analysis of genomic variations using data from multiple VZV open reading frames. The sequences of the strain Kawaguchi and the VZV isolate from the aqueous humor of this patient were referred to the published data. The sequence positions are based on the sequence of the European VZV strain Dumas (GenBank Accession no. NC_001348). The twenty-seven SNPs proposed by Breuer et al. [10] are in orange. Clade 2 markers are in yellow, Oka vaccine (v-Oka) strain markers are in red, and other clades markers are in green. ND, not discriminated

Additionally, the amplicons of ORFs 1, 6, 12, 16, 17, 21, 22, 35, 37, 50, 54, 55, 56, 60, 62, and 66 were sequenced as previously described [21, 23]. The 27 SNPs of these ORFs were previously proposed for the classification of VZV strains [10]. The informative polymorphic markers on the regions are shown in Fig. 5. Based on the nomenclature scheme proposed by Breuer et al. [10], the VZV isolate from the aqueous humor of this patient was grouped among the clade 2 strains, a genotype predominantly present in Japan and surrounding countries [4, 9]. In addition, we found three SNPs that differed from those of clade 2, in which one SNP (C at position 87841) was unique to clade 1, the dominant clade in Europe and North America.

## Discussion

We experienced a rare case of VZV-associated uveitis with severe hyphema in an immunocompetent boy without eruptions or vaccination history. The clinical findings, such as unilateral uveitis with elevated IOP, segmental iris atrophy, and distorted pupil, were compatible with the characteristics of VZV-associated uveitis [24]. A definitive diagnosis of ZSH can be made by detecting VZV-DNA in the aqueous humor [25]. In this case, because VZV-DNA was detected in the aqueous humor by multiplex PCR at presentation, we could make a definitive diagnosis and start treatment at an early stage. Quantitative real-time PCR revealed that a large number of VZV-DNA copies were present in the aqueous humor, which decreased and disappeared as ocular inflammation improved with acyclovir/famciclovir treatment.

Antibody examination is also useful in the diagnosis of ZSH [26]. In the present case, a gradual decrease was observed in serum anti-VZV IgG and serum anti-VZV IgM along with improvement in ocular inflammation, and these findings were also useful in the evaluation of therapeutic effects.

In most cases of uveitis with hyphema, the cause of bleeding is supposed to be rubeosis iridis [12]. In VZV-associated uveitis, iris fluorescein angiography suggests occlusive vasculitis to be the main cause of the iris lesion responsible for the bleeding [14, 27]. At the initial visit, the rubeosis iridis could not be observed due to severe hyphema and iris fluorescein angiography was not performed. Taking into account the fact that rubeosis iridis developed at 4 months after the initial visit, the hyphema may have been due to rubeosis iridis. Moreover, the marked iris atrophy observed after the resolution of inflammation suggests the presence of severe inflammation that may have caused obstruction of the iris vessels.

Genetic variations have been identified in the genome of VZV strains from vesicle fluid, varicella scabs or throat swab samples by molecular techniques [2–9]. However, there have been no reports on the genotyping of VZV using DNA extracted from the aqueous humor. It is generally considered that genetic analysis using the aqueous humor is difficult as we can only collect a small amount of aqueous humor and it contains only little or incomplete VZV genomes. Kido et al. reported that quantitative real-time PCR detected significant viral loads of VZV-DNA (ranging from 3.86 × 10^2^ to 1.26 × 10^7^ copies/mL) in the aqueous humor of eight patients with VZV-associated uveitis [25]. In this case, quantitative real-time PCR revealed that a very large number of VZV-DNA copies were present (1.61 × 10^9^ copies/mL) in the aqueous humor at presentation, which subsequently fell to 1.02 × 10^6^ copies/mL at 4 weeks after presentation. We could analyze the VZV genotype using DNA obtained at presentation, but not with DNA obtained at 4 weeks after presentation. This is a valuable report in terms of our ability to obtain a very large number of VZV-DNA copies and analyze VZV genotype using the aqueous humor. To the best of our knowledge, this is the first investigation of VZV genotype to use aqueous humor.

RFLPs analysis of VZV strains have identified a relationship between VZV genotype and climate. ORF38 (*Pst*I), ORF54 (*Bgl*I) and ORF62 (*Sma*I) in the VZV genes are molecular genetic markers for the genotyping of VZV strains [9, 28, 29]. Previous studies have shown that the absence of a *Pst*I restriction site (*Pst*I^−^) in ORF 38 and the presence of a *Bgl*I restriction site (*Bgl*I^+^) in ORF 54 differentiate v-Oka from wild-type American strains [28, 29], such as strain MLS, and European strains [6, 30], such as strain Dumas. The differences reflect geographical variations between the Japanese v-Oka and non-Japanese strains, but these markers do not distinguish v-Oka from its parent wild-type virus (p-Oka) or from other wild-type Japanese strains such as strain Kawaguchi [31]. By analyzing the *Sma*I restriction enzyme cleavage sites in ORF 62, which only v-Oka creates, v-Oka can be differentiated from p-Oka and other wild-type Japanese strains [8]. In the present study, the VZV isolates in the aqueous humor of this patient were *Pst*I^−^ in ORF38, *Bgl*I^+^ in ORF54, and *Sma*I^−^ in ORF 62 (Fig. 4 and Table 1). These results are consistent with the findings for wild-type Japanese VZV strains.

The R5 variable region in the VZV genes has also been shown to have geographic variations [6, 21, 30]. The R5A (359 bp) type is mainly found in Europe and North America, including strain Dumas, strain MLS, and strain Ellen. The R5B (471 bp) type is a major type in Japan, including strain v-Oka, p-Oka, and Kawaguchi. Unlike these Japanese strains, R5A was observed in the current patient (Fig.4d and Table 1).

With the development of whole-genome sequencing, a meeting, held in 2008, was organized to create a common system for VZV classification and nomenclature. Based on the phylogeny of VZVs, five major clades (1–5) and two provisional clades (VI and VII) were proposed [10]. Multiple ORFs required for this classification in VZV genes have been sequenced and analyzed in various areas of the world [2, 9, 21, 23]. By referring to the SNPs, the VZV strain from the aqueous humor of this patient could be grouped among clade 2 strains, a genotype commonly found in Japan and surrounding countries [4, 9, 10]. Additionally, we found three SNPs that differed from those of clade 2, in which one SNP (C at position 87841) was unique to clade 1 (Fig. 5). It is likely that this strain may represent a new subclade of clade 2.

As there have been no reports on the genotyping of VZV in uveitis using aqueous humor, it is unknown whether this genotype is specific to uveitis or whether it differs from varicella or herpes zoster. Despite the thorough investigation of genetic variability among the circulating strains of VZV in recent decades, it is not yet known whether VZV genotype is linked to differences in the pathogenesis and virulence of VZV strains. One of the important limitations to this report is that we were not able to analyze any other VZV-DNA sequences from this patient to which the aqueous humor genomes could be compared as VZV-DNA was not detected in the plasma and no other specimens, such as throat swabs, could be collected at presentation. Therefore, future studies are warranted to analyze VZV genotypes using the aqueous humor, as performed in this study, as well as other specimens where possible in a large number of uveitis cases.

## Conclusions

In conclusion, we report a rare case of ZSH with severe hyphema, which was diagnosed by multiplex PCR using the aqueous humor at an early stage. The measurement of VZV-DNA copy numbers by quantitative real-time PCR, as well as serum anti-VZV IgG and serum anti-VZV IgM was useful for the evaluation of improvement in ocular inflammation and therapeutic effects. As far as we know, this is the first report that we could amplify and analyze VZV genotype using the aqueous humor including a very large number of VZV-DNA copies (1.61 × 10^9^ copies/mL). Genetic analysis showed that the isolate from the aqueous humor of this patient was a wild-type clade 2 VZV strain (prevalent in Japan and surrounding countries) with R5A allele and one SNP unique to clade 1 (both are mainly found in Europe and North America).

## Abbreviations

HZO: Herpes zoster ophthalmicus
IOP: Intraocular pressure
OCT: Optical coherence tomography
ORF: Open reading frame
PCR: Polymerase chain reaction
p-Oka: Parental Oka
RFLP: Restriction fragment length polymorphism
SNP: Single nucleotide polymorphism
v-Oka: Oka vaccine
VZV: Varicella-zoster virus
ZSH: Zoster sine herpete.
